# Intraparenchymal metastases to the spleen from ovarian cancer: a case report

**DOI:** 10.1186/1752-1947-4-30

**Published:** 2010-01-29

**Authors:** Abdul A Ghani, Zubair A Hashmi, Daniel M Chase, Shonak B Patel, Daniel F Jones

**Affiliations:** 1 Department of General Surgery, 500 Gypsy Lane, Suite 200, Youngstown, OH 44505, USA; 2 Department of Pathology, 500 Gypsy Lane, Youngstown, OH 44505, USA

## Abstract

**Introduction:**

Splenic tumors are rare and present a diagnostic dilemma. Metastatic carcinoma to the spleen is unusual. Visceral metastases in patients with ovarian cancer represent hematogenous spread of the disease; capsular involvement resulting from serosal and peritoneal seeding is more common. We present a patient with intraparenchymal splenic metastasis from ovarian carcinoma. This case demonstrates a rare etiology of an intraparenchymal solid splenic mass.

**Case presentation:**

An 85-year-old woman presented with left upper quadrant pain. During her evaluation, a computed tomography scan revealed intraparenchymal splenic masses. An elective splenectomy was performed, during which ovarian cancer, which had not been revealed by the pre-operative computed tomography, was detected. There was no involvement of the splenic capsule by direct extension of the tumor, as is usually the case for ovarian cancer, but only intraparenchymal metastases. This mode of metastasis to the spleen has been described but is quite rare, and ovarian cancer presenting as a splenic mass is even more so.

**Conclusion:**

Splenic metastasis is a relatively rare event. It is often asymptomatic and is usually detected as part of multiorgan metastases. Symptomatic cases, though rare, do occur, and as in our patient, a thorough clinical evaluation is important to help direct the treatment plan. This case is a reminder to be cognizant of one of the less likely differential diagnoses of an intraparenchymal solid splenic mass.

## Introduction

Splenic tumors are rare and present a diagnostic dilemma. The differential diagnosis of splenic tumors includes hemangioma, lymphangioma, hamartoma, hemangiosarcoma, malignant lymphoma, and metastatic carcinoma.

Metastatic carcinoma to the spleen is unusual. Visceral metastases in patients with ovarian cancer represent hematogenous spread of the disease, and are present in 2% to 3% of patients [1]. Capsular involvement resulting from serosal and peritoneal seeding is much more common [2]. A review of the literature reveals that about half of reported splenectomies for ovarian cancer were performed at the time of primary cytoreduction, and the other half were done at secondary debulking. The large majority of these did not have parenchymal splenic involvement [3].

We present a rare case of intraparenchymal splenic metastasis from ovarian carcinoma.

## Case presentation

An 85-year-old woman originally presented to her primary care physician with vague pain in her left upper quadrant. She described it as a sharp, intermittent pain, not associated with meals. The pain was exacerbated by different body positions and by activity. She experienced loss of appetite and five pounds of weight loss. She had no significant past medical history, but had recently had both upper and lower endoscopies, which were normal.

**Figure 1:**
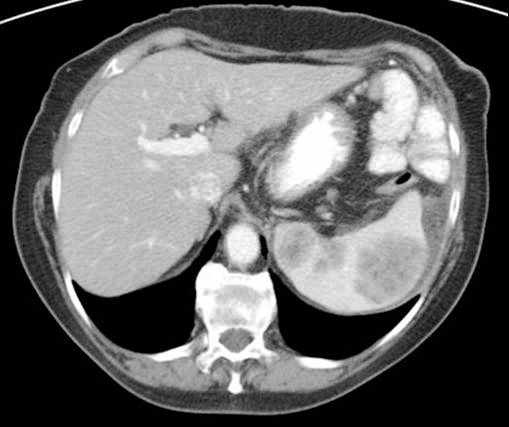
**Computed tomography scan showing splenic masses**.

Physical exam revealed tenderness in her left upper quadrant. No organomegaly was identified, and no other tenderness or palpable masses were present on examination. A computed tomography (CT) scan of her abdomen was ordered by her primary care physician, and this showed multiple masses within the spleen which were suspicious for malignancy (Figure 1). Because of the CT findings and the patient's complaint of pain, she was referred for splenectomy.

Pre-operatively, a complete blood count and electrolytes were within normal limits. A laparoscopic splenectomy was initiated but due to extensive adhesions and multiple tumor implants, the procedure was converted to an open approach. On exploration, numerous omental, mesenteric, peritoneal, and diaphragmatic implants were identified. The splenic capsule, however, appeared to be uninvolved. A splenectomy, omentectomy, removal of peritoneal nodules, and right oophorectomy were performed. The left ovary was not identifiable due to the dense adhesions and implants matted in that area.

**Figure 2:**
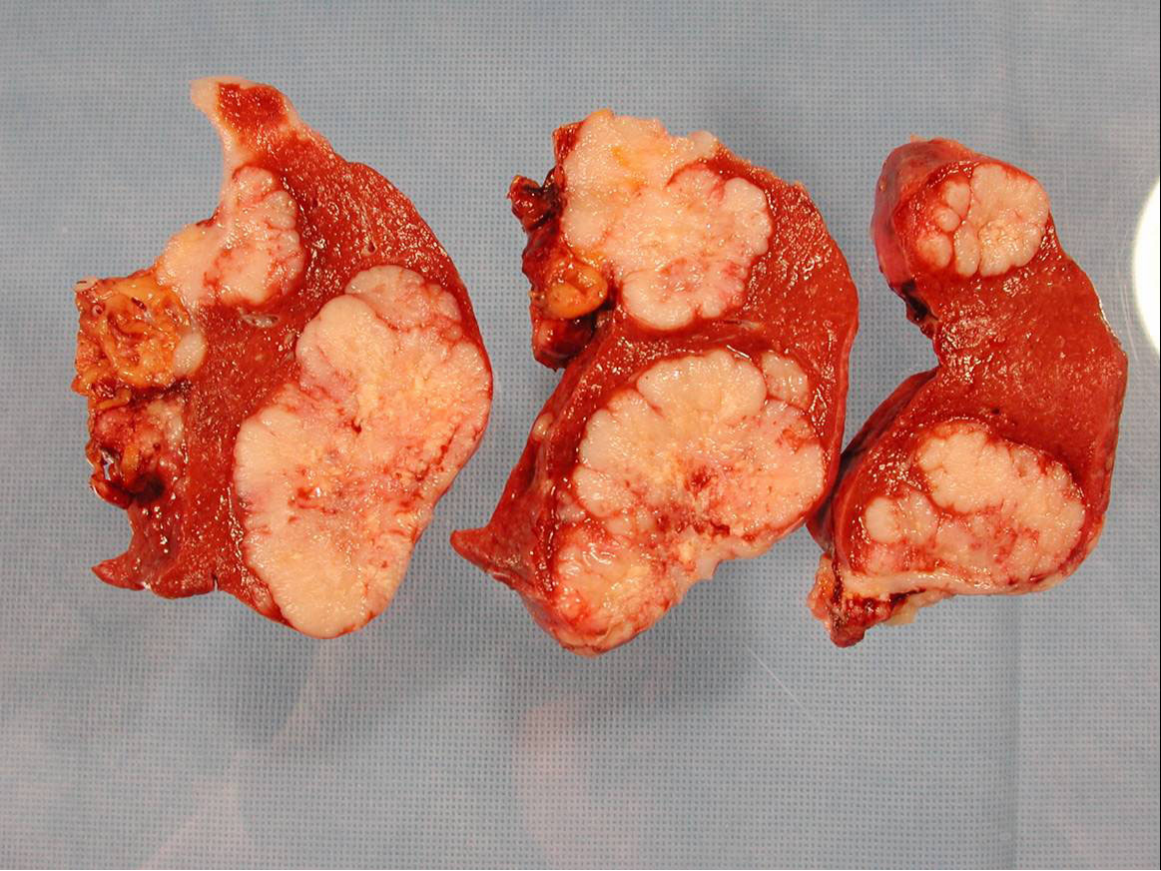
**Sections of spleen showing intraparenchymal masses**.

**Figure 3:**
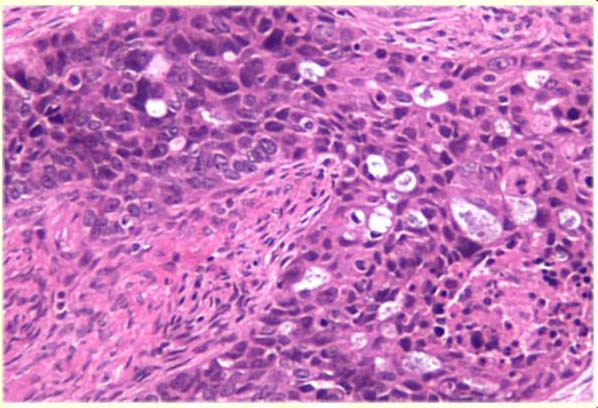
**Representative section of ovary stained with hematoxylin and eosin (100×) demonstrating irregular nests of large anaplastic cells with an ill-defined cribriform architecture infiltrating the ovary**. No residual normal ovarian tissue is shown.

**Figure 4:**
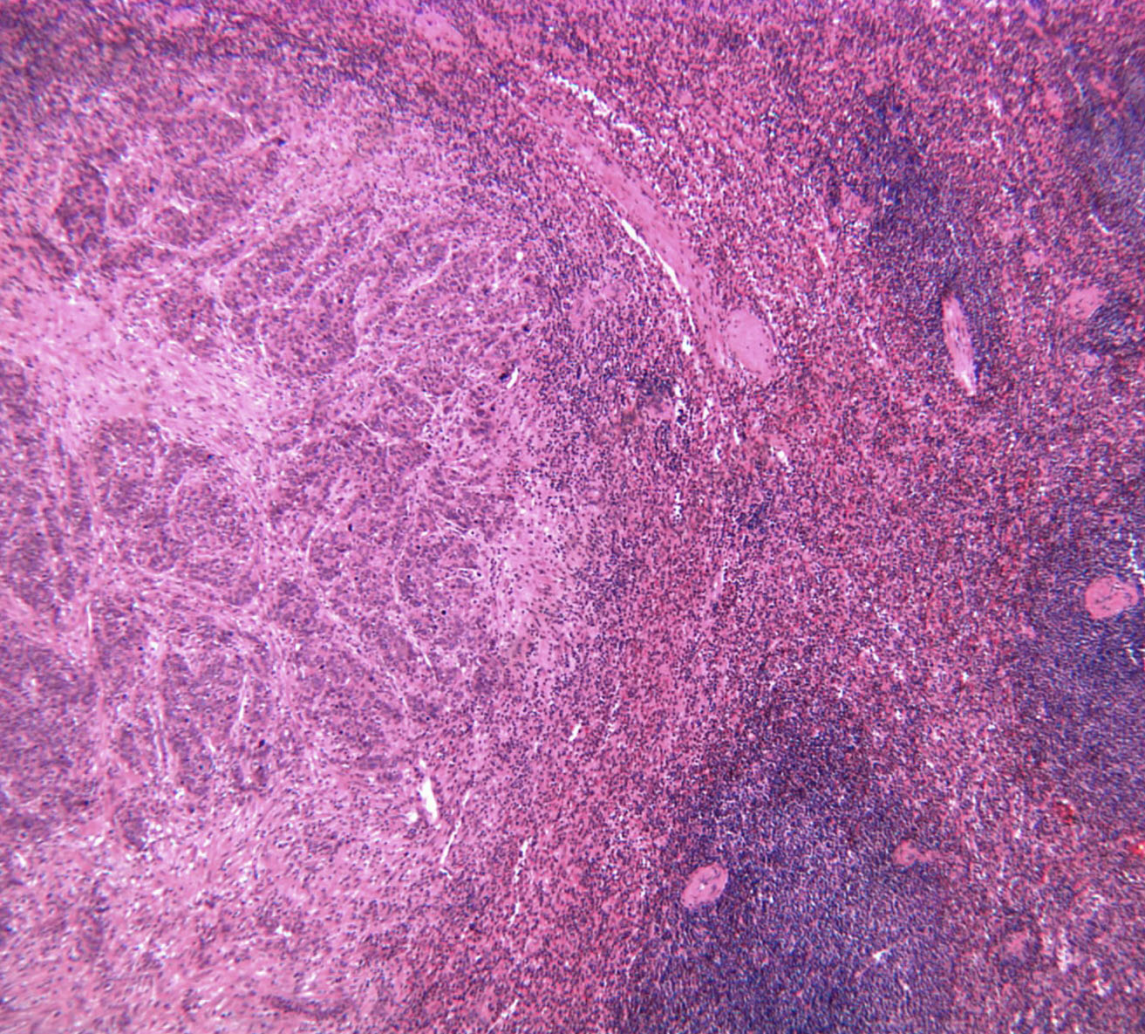
**Representative section of spleen stained with hematoxylin and eosin (20×) demonstrating morphology similar to that seen in the ovary**. Normal residual spleen is seen on the right.

Pathologic examination of the gross specimen (Figure 2) revealed intraparenchymal splenic lesions. Representative sections from the ovary (Figure 3) and spleen (Figure 4) are shown here. The tumor at both sites showed a similar morphology of a poorly differentiated adenocarcinoma consisting of irregular nests of large anaplastic polygonal cells with an ill-defined cribriform pattern (Figure 3, inset). The ovary was almost completely replaced by tumor. There were multiple splenic nodules showing invasive growth. Special stains (not shown) were identical between the sites, with small amounts of intracytoplasmic mucin on mucicarmine stain. Neoplastic cells showed positive immunohistochemical staining for pankeratin, estrogen receptor, and CK7 and were negative for progesterone receptor, carcinoembryonic antigen (CEA), and CK20 in sections from both sites (not shown). This immunophenotype is indicative of a primary ovarian adenocarcinoma.

A CA-125 level checked postoperatively was markedly elevated at 685.9 units/mL (normal: <35 units/mL). The patient made an uneventful postoperative recovery, and was discharged home. After a consultation with oncology, she is currently undergoing chemotherapy.

## Discussion

Metastatic carcinoma to the spleen is rare [1,3]. The incidence of splenic metastasis among patients with solid tumors has been reported to vary from 9% to 16% [4]. There appears to be no difference in incidence between men and women, but it is more frequently found in the elderly [5]. Cancers of the lung, breast, skin, ovary, colon, and stomach are the most common primary sites, with lung being the most frequent [6]. In most cases, the spleen is involved as part of a diffuse carcinomatosis [3], and splenic metastasis reflects widespread tumor dissemination. Warren and Davis reported that the incidence of metastases to the spleen ranged from 0.3% to 4.8%, based on autopsy reports. The paucity of afferent lymphatics to the splenic parenchyma and the motility of the organ might explain why it is a rare site of metastasis [7]. Other proposed explanations have included: the sharp angle made by the splenic artery, which makes it difficult for tumor emboli to enter the spleen; the rhythmic contractile nature of the spleen, which may squeeze out the tumor emboli; and antitumor activity due to a high concentration of lymphoid tissue in the spleen [5].

Lam and Tang conducted a 25-year-long clinicopathologic study where they concluded that metastatic disease involving the spleen may not be readily identified for several reasons. First, many splenic metastases are asymptomatic, making their detection more difficult. Symptomatic lesions tend to be larger, and thus are more likely to be part of a more widely metastatic tumor. Also, some patients in the study had splenic lesions that were so small that they could not be easily identified on gross examination during an abdominal exploration. Lastly, many of the grossly detectable lesions were solitary or diffuse rather than multiple, making them more likely to be confused with primary splenic tumors such as lymphomas or hamartomas [5].

The location of the primary tumor appears to affect the frequency of metastases to the spleen. For example, one series found that pancreatic primary tumors metastasized to the spleen more often than to other primary sites [5]. The proximity of the primary and the direct vascular access of the splenic artery and vein doubtless contributed to this finding. It has also been noted that types of tumors with a high mortality rate and high incidence within a given population are more likely to be the primary tumor in a patient with newly discovered splenic metastasis.

Late metastases to the spleen are seen more often in melanomas, choriocarcinomas, and breast carcinomas. Symptomatic lesions, when compared to asymptomatic lesions, were larger and were found more often in women and younger patients [5]. Those asymptomatic lesions that have been reported have been associated with splenic rupture [8]. Splenic rupture as a presentation of metastatic disease has been described, but is very rare [5]. In our patient, the splenic lesions were large and were easily seen on CT scan, and were probably contributing to the patient's left upper quadrant pain.

Metastases to the spleen are frequently found as part of a widely metastatic primary tumor. In one study, all patients found to have metastatic splenic tumors eventually died of complications of their primary tumors [5]. Splenectomy has been performed for metastatic disease for tissue diagnosis and for prevention of eventual splenic rupture. However, it has been proposed that routine resection of the primary tumor may not be justified based on the likely widespread nature of the tumor and high associated mortality rate [9].

Involvement of the splenic capsule in epithelial ovarian cancer usually occurs with widespread tumor dissemination [3], and is not as rare as metastatic parenchymal splenic involvement. Autopsy studies have revealed that splenic capsular involvement occurred in 19% to 52% of cases of epithelial ovarian cancer [10]. In most of these reports, the spleen was involved as part of diffuse carcinomatosis, and there are only a few cases of isolated parenchymal metastases [1,11,12]. More common primary tumor sites of splenic metastases are lung, breast, and skin affected with melanoma [13].

Pathology from our case revealed metastatic poorly differentiated adenocarcinoma from the splenic lesions and poorly differentiated papillary mucinous cystadenocarcinoma from the right ovary. There were several parenchymal metastases in the spleen, and intra-abdominal dissemination of the tumor. The capsule of the spleen in our patient was not involved by direct invasion. Parenchymal metastases may represent hematogenous spread of the disease, whereas capsular involvement represents peritoneal seeding [14].

Solitary splenic metastasis of carcinoma of ovarian origin is exceedingly rare, with fewer than 25 reported cases. Splenic involvement, even parenchymal involvement excluding direct invasion into the capsule, is usually associated with widespread intra-abdominal dissemination of the tumor. This, in fact, was the case with our patient, who had widespread visceral, peritoneal, and diaphragmatic tumor implants. In the rare cases of solitary splenic metastasis, splenectomy may have value beyond providing tissue diagnosis and preventing splenic rupture. Splenectomy, combined with oophorectomy and an appropriate chemotherapy regimen, can be part of a therapeutic and not merely a palliative procedure. Laparoscopic splenectomy, if achievable, is preferred over open splenectomy in these cases [2].

Our patient had multiple parenchymal splenic metastases and was symptomatic. In fact, her initial presentation was because of the discomfort caused by her affected spleen. She had an elevated CA-125 level and there were visible parenchymal splenic lesions on CT imaging. Splenectomy, omentectomy, removal of peritoneal nodules, and a right oophorectomy were performed. In reported cases, an open surgical splenectomy was performed except in two patients where a laparoscopic approach was used [2,3]. The advantages of laparoscopic splenectomy over an open approach include a shortened in-patient stay and a quicker recovery time. In general, patients who underwent a laparoscopic splenectomy can be started on a chemotherapy regimen sooner than those with an open splenectomy, which might favorably affect their outcome [2,13,15].

## Conclusion

In conclusion, splenic metastasis is a relatively rare event. It is often asymptomatic and is usually detected as part of multiorgan metastases [5]. Symptomatic cases, though rare, do occur, and as in our patient, a thorough clinical evaluation was important to help initiate the proper workup. This case is a reminder to be cognizant of one of the less likely differential diagnoses of an intraparenchymal solid splenic mass.

## Consent

Written informed consent was obtained from the patient for publication of this case report and any accompanying images. A copy of the written consent is available for review by the Editor-in-Chief of this journal.

## Competing interests

The authors declare that they have no competing interests.

## Authors' contributions

AG provided the case information, and was a major contributor to the discussion section of the paper. ZH interviewed the patient, reviewed the medical records and wrote the case presentation. DC provided major contributions to the case presentation and discussion sections, and edited the final manuscript. SP researched the subject and provided major contributions to the discussion section. DJ researched and wrote the pathology portion of the manuscript and prepared the histology figures. All authors read and approved the final manuscript.
